# Roles of Distal and Genic Methylation in the Development of Prostate Tumorigenesis Revealed by Genome-wide DNA Methylation Analysis

**DOI:** 10.1038/srep22051

**Published:** 2016-02-29

**Authors:** Yao Wang, Rohit Ramakant Jadhav, Joseph Liu, Desiree Wilson, Yidong Chen, Ian M. Thompson, Dean A. Troyer, Javier Hernandez, Huidong Shi, Robin J. Leach, Tim H.-M. Huang, Victor X. Jin

**Affiliations:** 1 Department of Molecular Medicine, The University of Texas Health Science Center at San Antonio, San Antonio, TX 78229, US; 2 Department of Cellular and Structural Biology, The University of Texas Health Science Center at San Antonio, San Antonio, TX 78229, US.; 3 Department of Epidemiology and Biostatistics, The University of Texas Health Science Center at San Antonio, San Antonio, TX 78229, US; 4 Department of Urology, The University of Texas Health Science Center at San Antonio, San Antonio, TX 78229, US; 5 Cancer Therapy and Research Center, The University of Texas Health Science Center at San Antonio, San Antonio, TX 78229, US; 6 Department of Pathology, The University of Texas Health Science Center at San Antonio, San Antonio, TX 78229, US; 7 Department of Biochemistry and Molecular Biology, Georgia Regents University, Augusta, GA 30912, US

## Abstract

Aberrant DNA methylation at promoters is often linked to tumorigenesis. But many aspects of DNA methylation remain unexplored, including the individual roles of distal and gene body methylation, as well as their collaborative roles with promoter methylation. Here we performed a MBD-seq analysis on prostate specimens classified into low, high, and very high risk group based on Gleason score and TNM stages. We identified gene sets with differential methylation regions (DMRs) in Distal, TSS, gene body and TES. To understand the collaborative roles, TSS was compared with the other three DMRs, resulted in 12 groups of genes with collaborative differential methylation patterns (CDMPs). We found several groups of genes that show opposite methylation patterns in Distal and Genic regions compared to TSS region, and in general they are differentially expressed genes (DEGs) in tumors in TCGA RNA-seq data. IPA (Ingenuity Pathway Analysis) reveals AR/TP53 signaling network to be a major signaling pathway, and survival analysis indicates genes subsets significantly associated with prostate cancer recurrence. Our results suggest that DNA methylation in Distal and Genic regions also plays critical roles in contributing to prostate tumorigenesis, and may act either positively or negatively with TSSs to alter gene regulation in tumors.

Prostate cancer (PCa) is the most common non-cutaneous cancer among men in the United States, and can be successfully treated if it is diagnosed early before metastasizing to bones or other organs. Over 240,000 men being diagnosed with prostate cancer in the US annually, a majority harbor local or regional disease where the long-term prognosis is excellent^1^. In contrast, the 5-year survival rate is only ~28% for the distant stage prostate cancer that already has metastasis in bones or other organs. Radical prostatectomy (RP), a surgical removal of all of the prostate gland, the seminal vesicles and the vas deferens, is one of the treatment options when the cancer is refined to the prostate. Nearly 40% of the patients undergoing RP present clinic-pathologic features associated with increased risk of clinical metastasis such as rising PSA (Prostate Specific Antigen, an indicator of risking cancer) , high Gleason score, seminal vesicle invasion or lymph node involvement^2^,^3^,^4^,^5^. By and large, these clinical features are used for determining risk group of PCa and there are various classification methods that are adopted by different medical organization and entities^6^. The great heterogeneity in prognoses of patient undergoing RP requires more effort in optimizing patient management to tailor proper treatments^7^. In this respect, the factors that have enhanced capacity to predict risk of metastasis, and death of PCa following RP are indeed crucial for the successful treatment and clinical outcome of this disease.

Not only Epigenetic mechanisms, including DNA methylation, are essential for normal development by regulating gene transcription involved with cell division or differentiation^8^, but also control a number of key processes including tumorigenesis. Many studies revealed that, in disease process such as cancer, abnormal DNA hypermethylation in the promoter regions of genes causes transcriptional silencing^9^. Recent studies show that gene body (genic) methylation also plays an important role in the alteration of gene expression^10^,^11^. There is a causal relationship between genic DNA methylation and gene expression, which indicates that methylation at genic regions could be a therapeutic target in cancer^12^. Distal regulatory elements are also subject to epigenetic modification and could be a key feature of the cancer epigenomes^13^,^14^.

Many aspects of the regulatory roles of DNA methylation are not fully understood. For instance, the collaborative roles among distal, gene body and promoter methylation besides their individual role remain unknown. It is worthwhile to put forth efforts in conducting such genome-wide methylation pattern analysis and understanding their impact on gene expression. On this basis, we conducted this study by applying a MBD-seq protocol previously established in our laboratory^15^ on a cohort of 32 PCa prostatectomy specimens, as well as 8 normal (N) and 12 tumor-adjacent specimens (ADJ). The grouping of patients were determined by applying widely used risk classification methods, and the final grouping included low risk (L), high risk (H) and very high risk (VH). The result in methylation study was correlated with the RNA-seq data from The Cancer Genome Atlas (TCGA). We sought to investigate the methylation pattern in the four genomic regions based on a gene structure, *i.e.*, 5-Distal region, TSS, Genic and TES, and to identify genes with differential methylations among tumor and normal samples. We further perform inter-correlation analysis on those identified differential DMRs between TSS region and the other three genomic regions to identify the CDMPs. Next, in order to elucidate the impact of DNA methylation on gene expression we correlated the significant CDMPs with gene expression values obtained from TCGA RNA-seq. IPA analysis on significantly differentially expressed genes in these three groups hinted the involvement of AR/TP53 related signaling network, and this pathway associated genes were further investigated in a survival analysis. To summarize, we demonstrate DNA methylation in distal and intragenic regions also plays critical roles in prostate tumorigenesis, and may act either positively or negatively with TSS to alter gene regulation in tumors. The identified gene clusters could be potential biomarkers that might be immensely helpful in the management of PCa patients particularly in the process of deciding proper treatment option at the time of RP.

## Results

### Identification of differential methylation in PCa patient data

**Figure 1:**
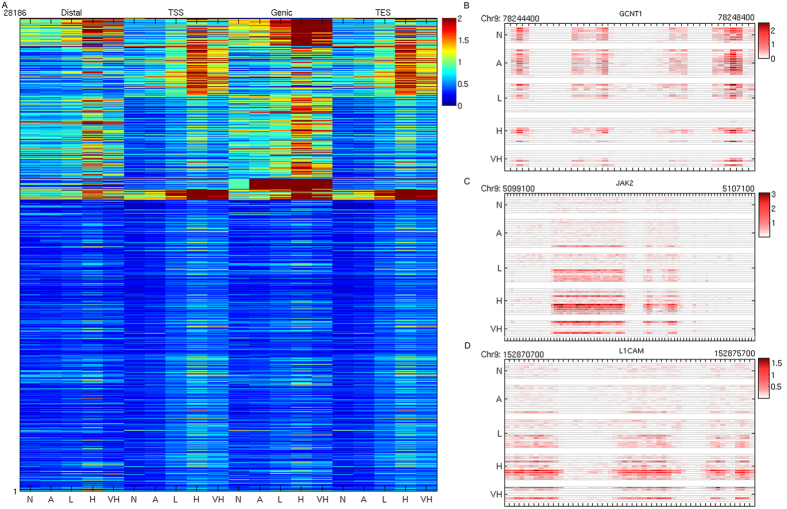
Differential methylation at a glance. (**A**) Unsupervised clustering of binned mean methylation by region. Each row represents the average methylation of one gene, and each column represents the average methylation of one sample group (N, A, L, H, VH), in one genomic region (Distal, TSS, Genic, TES). Columns are grouped by genomic region; (**B**) TSS region of gene GCNT1, shows hyper methylation in N and A group, comparing with H and VH risk group; (**C**) Genic region of Gene JAK2, shows hyper methylation in tumor group; (**D**) Distal region of gene L1CAM, shows hyper methylation in tumor group. Data in B–D are binned into 100 bp.

We applied a MBD-seq protocol previously established in our laboratory^15^ to investigate differential methylation patterns at a genome wide scale on a cohort of 32 PCa specimens, 12 tumor-adjacent specimens, 8 normal tissue specimens. Using LONUT^16^, we were able to utilize up to 95% of raw reads for most of samples with an average of 49.6 million of combined sequenced reads for all samples within five groups for the further analysis (Supplemental Table S2). First, for the four genomic regions, *i.e.*, Distal, TSS, Genic and TES (as described in Methods), we applied a unsupervised K-means clustering method to examine the mean methylation levels at a 100 bp bin-size resolution for five groups of samples in four genomic regions for all ~28,000 RefSeq genes (Fig. 1A). The results show that there are clearly unique methylation patterns for some genes in each of four genomic regions as well as multiple genomic regions. We also found that the methylation patterns in tumor adjacent samples are quite similar to the normal samples. Examples of different genomic regions harboring differential methylation are shown in Fig. 1B–D.

### Identification of collaborative methylation patterns

**Figure 2:**
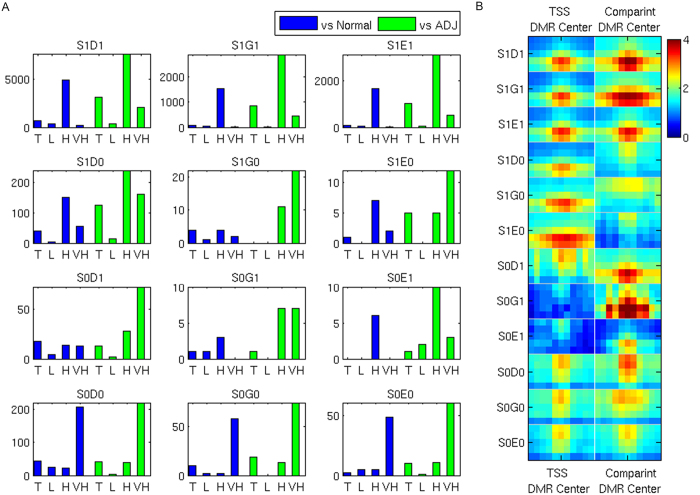
Differential methylation in each CDMPs. (**A**) Number of genes with DMRs in each of 12 groups; Blue bar represents the comparison between tumor vs normal samples; Green bar represents the comparison between tumor vs adjacent tumor samples; (**B**) Group Mean of each CDMPs, centered by middle point of region, extend to up and down 500 bp.

Albeit DNA hyper-methylation is expected to be exclusively found in tumor samples compared to normal counterparts, recent studies reveal frequent occurrence of DNA hypo-methylation co-existing with hyper-methylation in cancer^17^. In addition, many studies indicated that the methylation within gene body may be involved in altering gene expression in tumors^10^,^12^. Thus, it is very interesting to compare the collaborative methylation among different genomic regions in tumor with those in non-tumor samples. As a result, for each of the 12 distinct CDMPs as defined in Methods, we have tested the differential methylation between four tumor groups (all tumor, L, H, VH) and two non-tumor groups (N and ADJ), respectively. As in Fig. 2A, we observed that a significant number of genes have shown both hyper-methylations, i.e. tssHyper-distalHyper (S1D1), tssHyper-genicHyper (S1G1) and tssHyper-tesHyper (S1E1), while much less but still considerable number of genes have shown both hypo-methylation, i.e. tssHyper-distalHypo (S1D0), tssHyper-genicHypo (S1G0) and tssHyper-tesHypo (S1E0). Interestingly, we identified a certain number of genes showing opposite differential methylation in any two regions (e.g. hyper in TSS and hypo in Distal). In all, we found that: 1) dual hyper-methylations are the most common pattern identified in each of two-group comparison; 2) differential genes between tumor samples and adjacent samples are more than those between tumor samples and normal samples. 3) the number of genes in the other three patterns are far more less, from around one hundred in Distal compared to TSS, to just a dozen in TES compared to TSS region. We further plotted their mean methylation of the identified DMR for each set of genes (Fig. 2B); 4) high risk group shows the most instances of dual hyper-methylations in more than 5,000 genes, while very high risk group always have lower number of genes comparing with high risk group. This pattern is also extensively found in TSS compared to Genic or TES region; 5) low risk group samples almost always have the least number of genes with differential patterns; 6) very high risk group samples have the most number of DMR genes in dual hypo-methylation pattern.

### Correlation of gene expression from the RNA-seq data of prostate carcinoma in TCGA

**Figure 3:**
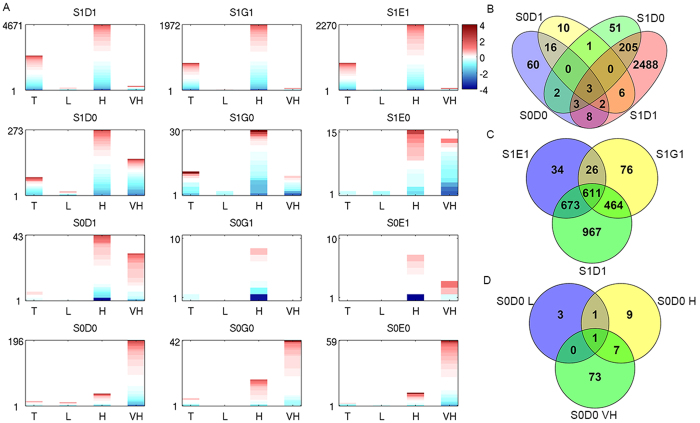
Correlating with TCGA RNA-seq data. (**A**) Log2 Ratio between tumor and normal samples, of genes that have positive log2 CPM and are identified with DMR; (**B**) Venn diagram of DEGs belonging to S1D1/S1D0/S0D1/S0D0 ; (**C**) Venn diagram of DEGs belonging to S1D1/S1G1/S1E1; (**D**) Venn diagram of DEGs in S1D1, among different risk groups.

To understand whether the identified distinct CDMPs have influenced the gene expression, we utilized the level-3 RNA-seq data of prostate carcinoma from TCGA to correlate with the identified genes associated with CDMPs. TCGA RNA-seq fold changes of all genes with DMR in each risk group and DMR are shown in Fig. 3A.

Overall, high risk group samples consist of more DEGs than the other three cancer groups, except in the events of dual hypomethylation pattern which are more prevalent in very high risk group. Among the 12 distinct CDMPs, S1D1, S1G1 and S1E1 groups have thousands of DEGs. For the other nine groups, the number ranges from several to several hundreds. For each of the 12 distinct CDMPs, we summarized the genes that are differential in any of the four tumor groups comparing with the two non-tumor groups as defined in the last section. Then, we compared and examined the DEGs to see whether their associated CDMPs are common or unique in TSS compared to one of the other three genomic regions.

1) TSS compared to Distal: Among all those genes showing both hyper-methylations in TSS and Distal region (S1D1), 2,715 genes are differentially expressed (1,210 up-regulated and 1,505 down-regulated). In contrast, only 94 differentially expressed genes are in both hypo-methylation pattern in these two regions (S0D0), including 57 up-regulated and 37 down-regulated. Similarly, 265 genes from the group with hyper-methylation in TSS and hypo DMR in Distal area (S1D0), 102 genes were up-regulated with concomitant down-modulation of 163 genes. Lastly, the group with hypo-methylated TSS and hyper Distal DMR has the smallest set of 38 genes (S0D1), 27 up and 11 down, respectively. Of note, L1CAM, found in S0D0 and S0D1 group, is expressed in androgen-insensitive and highly metastatic PCa cell lines, and is associated with PCa metastasis^18^. The two CDMPs of TSS hyper, i.e. S1D1 and S1D0 are significantly overlapped (hypergeometric distribution p-value = 8.17e-164); the two CDMPs of TSS hypo, i.e. S0D1 and S0D0 are also significantly overlapped (p-value = 2.32e-43); there is no significant overlap between S1D1 and S0D0, or S1D1 and S0D1 (Fig. 3B). 2) TSS compared to Genic: of 1177 genes showing differentially expression in TCGA data and having both TSS hyper and Genic hyper- DMRs (S1G1), 524 of them are surprisingly up-regulated. Since hypermethylation in promoter regions are often associated transcription silencing, we speculate that these genes might be transcriptionally controlled by gene body methylation resulting in up-regulation despite of the promoter hypermethylation. Similarly, S1G1 and S1G0 are overlapped with a p-value of 1.76e-08. However, there is no significant overlap between any other two CDMPs. 3) TSS compared to TES: There are a total of 1344 DEGs showing both hyper TSS and TES DMRs (S1E1), including 692 up and 652 down-regulated.

Furthermore, we looked at the overlapping DEGs among different CDMPs. Generally, the three both hyper-methylation CDMPs, i.e. S1D1/S1G1/S1E1, have significant overlap (Fig. 3C). Similarly, the three both hypo-methylation CDMPs, i.e. S0D0/S0G0/S0E0 also have significant overlaps. This indicated that hyper-methylation in TSS region are likely to be accompanied by hyper-methylation in distal, genic as well as TES region, and the same for the scenario of hypo methylation in TSS region.

Finally, we categorized genes that are only differentially expressed in one specific risk group (Supplemental Fig. S1). For example, in S0E0 there are 25 genes that are recognized as DEG only in VH risk group, and in S0D0 there are 73 genes that are only in VH risk group (Fig. 3D). We are particularly interested in genes that are only differential in very high risk group, as these genes are potentially associated with cancer progress and metastasis. These genes will be further investigated in survival analysis.

### Signaling pathway and network analysis

**Figure 4:**
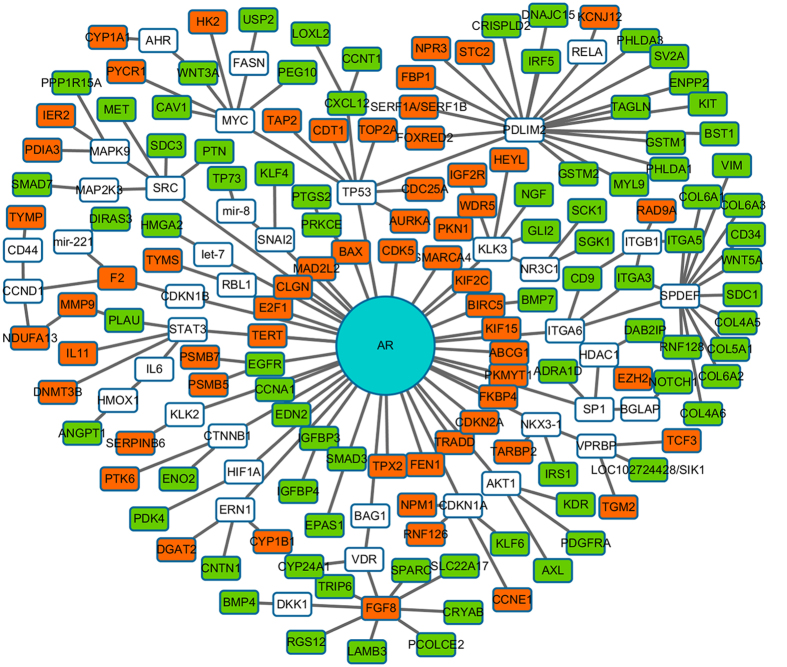
Network analysis results by IPA carried on gene sets that are differential in TCGA RNA-seq data. IPA generated pathways were trimmed to only keep the differential expressed genes that are directly or indirectly connected to AR, then visualized by Cytoscape.

For these 12 groups of genes showing both differential methylation and gene expression, we selected seven groups with more than 25 genes in each group and carried out network analysis using Ingenuity Pathway Analysis (IPA). Four groups, S1D1, S1D0, S1G1 and S1E1, have been shown to be biologically significant and linked to various pathways and networks. For example, the group SID1 with most number of genes has been identified to be associated with nine sub-networks, including cancer, cell death and survival, cellular development and proliferation, as well as DNA replication, recombination and repair. With only difference in a few genes from the group S1D1, the networks of both groups S1G1 and SIE1 are almost included by the network of S1D1. The pathway illustration by IPA was trimmed and visualized by Cytoscape^19^ as in Fig. 4. The top canonical pathways of each group are shown in Supplemental Fig. S2–S8.

Through a detailed examination of those IPA analyses, the following interesting results have emerged: 1) Common top ranked pathways: There are several canonical pathways that are commonly found, including G-protein coupled receptor signaling (GPCR), Gαi signaling, Thrombin signaling, cAMP-mediated signaling, CREB signaling in neurons, CXCR4 signaling, role of NFAT in cardiac hypertrophy and α-Adrenergic signaling. GPCR signaling^20^ is known to regulate cellular motility, growth and differentiation, and gene transcription. CXCR4 signaling, a member of GPCR family, is also extensively involved in tumor progression, angiogenesis, metastasis, and survival^21^. Gαi has been closely associated with CXCR4 signaling by assisting the mediation of CXCR4^21^. PDE4D7, an enzyme related to cAMP-mediated signaling pathway, was reported to be down regulated in AR-independent PCa cells and mediating proliferation^22^. CREB, responding to hormonal stimulation of the cAMP pathway, is associated with AR in cancer cells. Nonetheless, Thrombin signaling is reported to contribute to more malignant phenotype by activating tumor growth and metastasis^23^, and overexpression of relaxin is associated with accelerated progression of PCa^24^. NFAT proteins are functional in tumor cells during carcinoma progression and impact cell growth, survival, invasion and angiogenesis^25^. 2) S1D1-specific pathways: In addition to the common pathways, S1D1 group has a few specific canonical pathways, including gamma-glutamyl cycle, whereas gamma-glutamylcyclotransferase is a promising diagnostic marker and therapeutic target for prostate and various cancers^26^. Granulocyte adhesion and diapedesis pathway was reported to be found significantly enriched in a recent study on mammary cancer development^27^. There is also protein kinase a (PKA) signaling, which is functionally linked to AR in the progression of PCa^28^. 3) S1G1-specific pathways: S1G1 group also shows additional enriched pathways including cardiac β-adrenergic signaling, IL-1 signaling, P2Y purigenic receptor signaling, and PKA signaling. β-adrenergic signaling participate in multiple cellular processes that contribute to the initiation and progression of cancer, and is associated with cAMP signaling^29^. Dysregulated activation of the IL-1 signaling pathway contributes to cancer progression by creating highly inflammatory environment^30^. P2Y2 receptor promotes cell invasion and metastasis in PCa cells^31^. 4) S1E1-specific pathways: There are several pathways that are only found in S1E1 group, including Urea cycle which had been found to be strongly upregulated in basal subtype of triple-negative breast cancer^32^.

### Gene function and survival Analysis

We further performed Go Ontology (GO) functional analysis on a total of 139 differentially expressed genes from the IPA analysis (Supplemental Tables S4 and S5) using the DAVID tool^33^,^34^ and obtained several GO terms clusters related to developmental process, cell differentiation, regulation of apoptosis, regulation localization, cell aging, cell motility, and others that are crucial in cancer development and metastasis. Many of those genes have been reported as gene signatures or important genes in PCa. For example, CDKN1A is reported as one of a two-gene signature, which could distinguish indolent prostate tumor from aggressive tumor, and accurately predict outcome of low Gleason score prostate tumors^35^, the other is able to predict survival of castration-resistant PCa^36^. A mouse model revealed that Axl is an essential regulator of PCa proliferation and tumor growth^37^. COL6A2 was identified as a member of clinically relevant androgen-dependent gene signature in PCa^38^. CXCL12 is known to interact with CXCR4 in modulating PCa cell migration, metalloproteinase expression and invasion^39^. DAB2IP is a unique intrinsic AR modulator in normal cells, and likely can be further developed into a therapeutic agent for PCa^40^. E2F1 was proved to be associated with androgen-dependent growth, differentiation and apoptosis of PCa cells^41^. Down-regulation of MYL9 in stroma predicts malignant progression and poor biochemical recurrence-free survival in PCa^42^. PHLDA3 is identified as diagnostic and progression biomarkers of PCa^43^. SMAD3 inhibition rescues cancer cell proliferation in PC3 cells^44^. TYMS is associated with aggressive tumor features and early PSA recurrence in PCa^45^. Although several genes, to our knowledge, haven’t yet reported to be directly associated with PCa, we postulate that these genes could be potential biomarkers for PCa.

**Figure 5:**
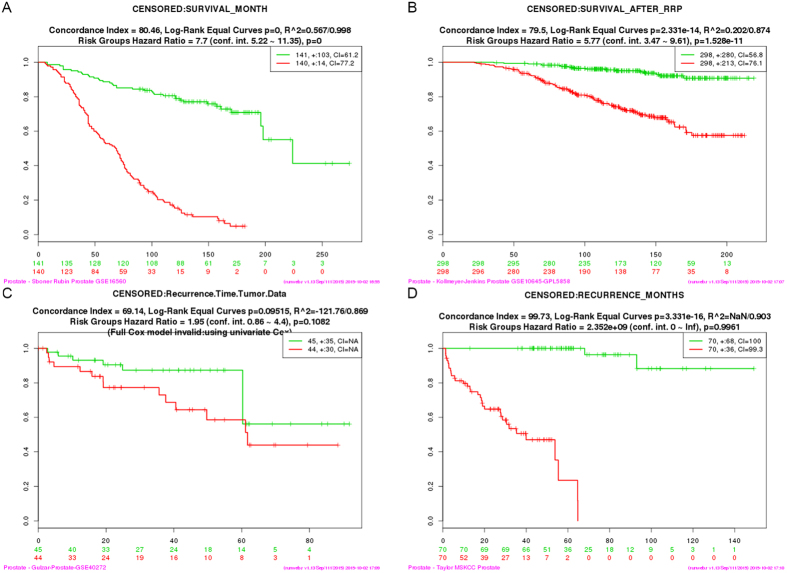
Survival analysis on public PCa data available by SurvExpress. (**A**) Survival by month on Sboner Rubin PCa data; (**B**) survival after RRP on Kollmeyer Jerkins PCa data; (**C**) recurrence on Gulzar PCa data; (**D**) recurrence on Taylor PCa data.

To further examine the genes that constitute the top ranked pathways identified by IPA analysis, we used SurvExpress^46^ as described in Methods. The Sboner Rubin (Fig. 5A) and Kollmeyer-Jenkins (Fig. 5B) Prostate datasets demonstrated significant association with patient survival. Gulzar data set also indicates clear association with disease recurrence (Fig. 5C). Analysis on Taylor data sets indicates that they are significantly associated with PCa recurrence (Fig. 5D).

For those genes that are only differentially expressed in very high risk tumor group (see Supplemental Table S6 for the list of genes), we also conducted survival analysis on Taylor MSKCC Prostate dataset (see Supplemental Fig S9). These genes were also significantly associated with PCa metastasis.

## Discussion

Despite the notion that DNA promoter hyper-methylation is linked to tumorigenesis is well established^9^, the roles of differential methylation (either hyper or hypo) in distal or gene body haven’t been fully examined. Through an integrative genomic analysis on a cohort of 52 PCa samples and non-tumor control, with tumor tissue samples being classified into three risk groups (low risk, high risk and very high risk), our study reveals individual roles of distal and genic methylation as well as their collaborative roles with promoter in contributing to prostate tumorigenesis. Interestingly, we observed extensive and intensive tumor hyper-methylations occurring in Distal, Genic, and TES regions in additional to in Promoter region (Fig. 1A). Although positively correlated hyper-methylation, *i.e.* TSS and Distal (S1D1), TSS and Genic (S1G1), TSS and TES (S1E1) is shown as a dominant pattern, we found other interesting collaborative differential methylation patterns (CDMPs) in this study (Fig. 2A). For example, there are a significant number of genes associated with TSS hyper-methylation and Distal hypo-methylation (S1D0) or both TSS and Distal hypo-methylation (S0D0). Given that very limited study has been focused on this aspect, it is worthwhile to further mechanistically interrogate how this opposite (gain or loss) methylation is processed during the tumor progression in the future study. Nevertheless, our current study, for the first time, provides a catalog of many newly discovered CDMPs for prostate tumors.

Another surprising observation emerges from our study is that down-regulation of gene expression is not prevalent among these genes associated with many different CDMPs (Fig. 3A). For example, of a total of 2851 DEGs for the combined S1D1, S1G1 and S1E1 groups, more than 40% (1250 genes in total) are noted to be up-regulated. These data suggest that gaining methylation in Distal, Genic or TES region might play similarly pivotal roles as those at promoters in impacting aberrant gene expression in prostate tumorigenesis. Despite several recent genome-wide studies implicate the roles of distal or intragenic methylation with cell or tissue type specificity or normal or stem cell developmental process^12^,^13^,^14^, very few studies have been conducted at a genome-wide scale to interrogate their roles in cancer stage specificity or cancer progression. By and large, our study provides a first genome-wide analysis on the specificity of PCa risk group.

Notably, our *in silico* IPA analysis hints that many common top ranked pathways are shared among three promoter-centered hyper-methylation groups (S1D1, S1G1 and S1E1), with androgen receptor (AR)-centered signaling network being predominant. Despite the clear role of AR signaling in PCa, our result for the first time suggests a potential functional link between a central regulatory role of AR and DNA methylation in PCa progression. Undoubtedly, further experimental studies are essentially needed for functional validation of such observation by deploying the approach of reverse genetics.

The DAVID GO analysis not only confirms many GO terms have been identified in the IPA analysis, but also reveals many of those genes previously implicated as gene signatures or important players in the progression of PCa. Remarkably, survival analysis indicates these particular sets of genes as significantly enhanced risk factors in PCa progression and patient survival (Fig. 5). We predict that these set of genes might be potential biomarkers in the process of determining treatment strategy for patient undergone RP for better clinical outcomes.

PCa is the most common cancer among men but could be the least life-threatening if receives proper treatment. However, the current prevailing clinical screening or testing approach suffers from limitations because of the inability to manage patients particularly with respect to prevent over or insufficient treatments. Our genome-wide study for the first time provides thousands of differential methylation regions as well as the genes associated with many different collaborative differential methylation patterns for PCa. Furthermore, our work provides insight into how DNA methylation in Distal and Genic regions might play critical roles in contributing to prostate tumorigenesis and henceforth may act either positively or negatively with TSS to alter gene regulation in tumors.

## Material and Methods

### DNA samples

DNA samples isolated from 32 prostate tumors, 12 tumor adjacent normal tissues, 8 normal prostate tissues (Supplementary Table S1) were subjected to subsequent DNA methylation analysis. The prostate tumor samples were collected from patients at different stages of tumor advancement while normal prostate tissues were obtained from healthy individuals. Informed consent was obtained from patient according to IRB protocols approved by the University of Texas Health Science Center at San Antonio and the University of Manitoba at Winnipeg, respectively. All experiments were performed in accordance with approved guidelines of the Institutional Review Board committee at UTHSCSA.

### MBDCap sequencing (MBD-seq)

Methylated DNA was eluted by the MethylMiner Methylated DNA Enrichment Kit (Invitrogen) according to the manufacturer’s instructions. Briefly, one microgram of genomic DNA was sonicated and captured by MBD proteins. The methylated DNA was eluted in 1 M salt buffer. DNA in each eluted fraction was precipitated by glycogen, sodium acetate and ethanol, and was resuspended in TE buffer. Eluted DNA was used to generate libraries following the standard protocols from Illumina. Next, MBDCap-seq libraries were sequenced using the Illumina Genome Analyzer II as per manufacturer’s instructions. Image analysis and base calling were performed with the standard Illumina pipeline.

### Bioinformatics analysis of MBD-seq data

Single-end 50 bp reads were mapped to the UCSC human transcriptome (hg18) by Bowtie with parameters as -v 2 -best -k 10. Multiple matched reads were processed by LONUT^16^, a computational tool for locating multiple-matched reads in order to improve the detection of the enriched regions for ChIP-seq and MBD-seq data. We retained the multiple matched reads which were in proximity to peaks detected in the uniquely matched reads and combined them with uniquely matched. The combined reads were then binned by 100 bp bin-size and normalized by total reads of each sample. A detailed description of each individual MBD-seq data is in Supplementary Table S2.

For risk group classification of samples, we combined the result from four methods that were discussed previously^6^, as defined by American Urological Association, European Association of Urology, Radiation Therapy Oncology Group and National Comprehensive Cancer Network , respectively. For each patient sample, the highest risk among the four methods was assigned. Therefore, the total 32 tumor samples were split into three risk groups: 13 low risk, 12 high risk, and 7 very high risk (Supplemental Table S1). In order to compare the tumor adjacent normal tissues with healthy normal tissues, we selected 12 patients’ adjacent tissues (ADJ) as a separate group, hence resulting in five different groups for the initial analysis.

For each transcript in UCSC RefSeq database that has unique transcription start site (TSS) and termination site (TTS), it is further divided into four genomic regions by the following manner: Distal (upstream 2–100 Kb), TSS (upstream and downstream 2 Kb of 5′), TTS (upstream and downstream 1 Kb of 3′), and Genic region (down 2 Kb of 5′ to up 1 Kb of 3′). The reads within the four genomic regions were then used for the differential methylation analysis. Differential methylation level at each bin (100 bp) was determined by a rank sum test between each tumor group and non-tumor group. For example, for Low risk samples, we compared them with normal samples, adjacent samples and normal combined with adjacent samples, respectively. We also combined all tumor samples together and did the same comparison with non-tumor samples. For TSS, TES and Distal regions, a minimum of three consecutive bins with a P-value less than 0.05, a minimum of 0.2 rpm on average in the higher methylated group, and a minimum log2FC of 1 must be considered for a statistically significant differential methylation region (DMR). However, a minimum of five consecutive bins is required for a Genic region.

We examined the collaborative methylation among different genomic regions in tumor compared to normal samples. Ideally, there should be 24 collaborative differential methylation patterns (CDMPs) based on any combination of two genomic regions associated with the same gene: both hyper-, both hypo-, hyper-/hypo- and hypo-/hyper. Considering that the DNA methylation status in a TSS region is critical in regulating tumorigenesis, to simplify the number of distinct CDMPs, in this study we only focused on those 12 patterns involved in a TSS region, i.e., S1D1 (hyper-TSS and hyper-Distal), S1G1 (hyper-TSS and hyper-Genic), S1E1 (hyper-TSS and hyper-TES), S1D0 (hyper-TSS and hypo-Distal), S1G0 (hyper-TSS and hypo-Genic), S1E0 (hyper-TSS and hypo-TES), S0D1 (hypo-TSS and hyper-Distal), S0G1 (hypo-TSS and hyper-Genic), S0E1 (hypo-TSS and hyper-TES), S0D0 (hypo-TSS and hypo-Distal), S0G0 (hypo-TSS and hypo-Genic), S0E0 (hypo-TSS and hypo-TES). We divided each genomic region into 100 bp bins and applied a rank sum test on each individual bin between each of three cancer patients (low, high, very high) groups and tumor adjacent normal (ADJ) or healthy normal (NORM) groups.

### TCGA data and analyses

RNA-seq datasets in PCa were downloaded from the Cancer Genome Atlas (TCGA) data portal (http://tcga-data.nci.nih.gov). We extracted level-3 raw count of genes using the “data matrix” tool provided by TCGA data portal. We selected a total of 523 samples, 52 normal and 471 tumor samples, available with both RNA-seq data and clinical features information for performing the correlation analysis. The 471 tumor samples consist of 23 low risk, 252 high risk and 196 very high risk samples. Differential was calculated using the edgeR package (version 3.4.2)^47^ in BioConductor (release 2.13, R version 3.0.2). Genes with <0 cpm in more than 150 of the samples were excluded, and genes with a P-value lower than 0.05 as well as log2FC greater than 0.5 were assigned as being differentially expressed. Venn diagram was drawn using online tool Venny^48^.

### Correlating result from TCGA data with MBD-seq data

Since the TCGA data do not have adjacent samples, for each CDMP and risk group, a gene is marked as with DMR if it is differential comparing with normal or adjacent. Besides, the reference genes and level-3 RNA-seq genes are not mutually inclusive. Therefore, we only kept the overlap genes between the two types of data. A heatmap of TCGA RNA-seq fold changes of all overlapping genes in each risk group and DMR are shown in Fig. 3A.

### Survival and IPA analyses

In the IPA analysis, genes that are differential in either all tumor vs normal, or tumor subgroups vs normal, were separated into list according to the methylation pattern and used as input to search for canonical pathways and networks. The number of genes used in IPA analysis is in Supplemental Table S3.

SurvExpress^46^, an online biomarker validation tool and database, was used to explore the patient survival or regression outcome. The tool takes gene list as input, as well as additional options for configuration, such as censoring, number of risk groups, stratification method, etc., to generate figures including Kaplan-Meier curves and gene expression heatmap. In our case, there are seven Prostate databases available, and we selected four datasets with the most number of samples, including 140-sample Taylor MSKCC Prostate^49^, 281-sample Sboner Rubin Prostate GSE16560^50^, 98-sample Gulzar Prostate GSE40272 51, and 596-sample Kollmeyer-Jenkins Prostate GSE10645-GPL5858 52. Since some of these data sets do not have the whole genome’s gene expression, the survival analysis was only performed on a part of the candidate genes from IPA analysis. We used the default setting of SurvExpress so that samples would be divided into two risk groups based on the quantile normalized expression in each data set, and there is no stratification.

## Additional Information

**How to cite this article**: Wang, Y. *et al.* Roles of Distal and Genic Methylation in the Development of Prostate Tumorigenesis Revealed by Genome-wide DNA Methylation Analysis. *Sci. Rep.* **6**, 22051; doi: 10.1038/srep22051 (2016).

## Supplementary Material

Supplementary Information
