# Supplementary material for: Roles of Distal and Genic Methylation in the Development of Prostate Tumorigenesis Revealed by Genome-wide DNA Methylation Analysis
Source: Sci Rep. 2016 Feb 29;6:22051. doi: 10.1038/srep22051 (PMC4770430; doi:10.1038/srep22051)
Supplement: Supplementary Information [file srep22051-s1.pdf]

Supplementary Information

Roles of Distal and Genic Methylation in the Development of Prostate Tumorigenesis Revealed by Genome-wide DNA Methylation Analysis

Yao Vickie Wang[1], Rohit Ramakant Jadhav[1], Joseph Liu[1], Yidong Chen[2], Ian M. Thompson[3,4], Dean A. Troyer[5], Javier Hernandez[3], Huidong Shi[6], Robin J. Leach[3,4,7], Tim H-M Huang[1,4], and Victor X Jin[1,2]

[1]Department of Molecular Medicine, [2]Department of Epidemiology and Biostatistics, [3]Department of Urology, [4]Cancer Therapy and Research Center, [5]Department of Pathology, [7]Department of Cellular and Structural Biology, The University of Texas Health Science Center at San Antonio, San Antonio, TX 78229; [6]Department of Biochemistry and Molecular Biology, Georgia Regents University, Augusta, GA 30912

Corresponding author:

Dr. Victor X Jin, Department of Molecular Medicine, University of Texas Health Science Center, 8403 Floyd Curl, San Antonio, TX 78229. Tel: 210-5629209; Email: jinv@uthscsa.edu

Table of Contents

Supplemental Table S1. Patient information of prostate cancer cohort.. 3
Supplemental Table S2. MBD-Seq data information 5
Supplemental Table S3. Number of genes used in IPA analysis 7
Supplemental Table S4. Genes shown in the network in Figure 4 that are also up regulated in TCGA data 7
Supplemental Table S5. Genes shown in the network in Figure 4 that are also down regulated in TCGA data 8
Supplemental Table S6. Genes that are only in very high risk group and also differentially methylated in cohort as well as differentially expressed in TCGA RNA-seq data 9
Supplemental Figure S1. Venn diagram of differential expressed genes between different risk group in each CMP 10
Supplemental Figure S2. S0D0 Canonical Pathways 10
Supplemental Figure S3. S0D1 Canonical Pathways 11
Supplemental Figure S4. S0E0 Canonical Pathways.. 11
Supplemental Figure S5. S1D0 Canonical Pathways 11
Supplemental Figure S6. S1D1 Canonical Pathways 12
Supplemental Figure S7. S1G1 Canonical Pathways 13
Supplemental Figure S8. S1E1 Canonical Pathways.. 14
Supplemental Figure S9. Kaplan Meier curve of genes that are only differentially expressed and methylated in very high risk group 15

Supplemental Table S1. Patient information of prostate cancer cohort

	Patient ID	Group	Age	Gleason Score	PSA	T Stage	N stage	M stage
1	HG_LCH_01	Normal	73	–	–	–	–	–
2	HG_LCH_04	Normal	66	–	–	–	–	–
3	HG_LCH_05	Normal	58	–	–	–	–	–
4	HG_LCH_06	Normal	80	–	–	–	–	–
5	HG_LCH_09	Normal	77	–	–	–	–	–
6	HG_LCG_10	Normal	66	–	–	–	–	–
7	HG_LCH_11	Normal	75	–	–	–	–	–
8	HG_LCH_12	Normal	63	–	–	–	–	–
9	HG_Lch_21	Low	68	6	2.37	T2B	N0	Mx
10	HG_Lch_22	Low	60	5	7.4	T2A	Nx	Mx
11	HG_Lch_23	Low	60	7	4.4	T2B	N0	Mx
12	HG_Lch_24	Low	50	6	2.8	T2b	Nx	Mx
13	HG_Lch_25	Low	69	8	6.8	T2B	Nx	Mx
14	HG_Lch_26	Low	56	7	9.36	T2B	Nx	Mx
15	HG_Lch_27	Low	68	6	6.27	Tx	Nx	Mx
16	HG_Lch_28	Low	69	7	1.8	T2b	N0	Mx
17	HG_Lch_29	Low	64	7	1.6	T2b	N0	Mx
18	HG_Lch_30	Low	65	6	9.25	T2a	N0	Mx
19	HG_Lch_31	Low	68	7	4.29	T2a	N0	Mx
20	HG_Lch_32	Low	63	7	8.65	T2b	Nx	Mx
21	HG_Lch_35	High	70	8	4.13	T2c	Nx	Mx
22	HG_Lch_36	High	60	7	4.21	T3	Nx	Mx
23	HG_Lch_38	VeryHigh	66	8	6.1	T3a	Nx	Mx
24	HG_Lch_39	VeryHigh	56	8	11.38	T3b	N0	Mx
25	HG_Lch_42	VeryHigh	63	9	11.24	T3b	N0	Mx
26	HG_Lch_45	High	57	7	8.62	T3	N0	Mx
27	HG_Lch_47	Low	64	7	N/A	T2b	Nx	Mx
28	HG_Lch_48	VeryHigh	70	10	8.79	T4	N1	Mx
29	HG_Lch_49	High	57	7	2.32	T3	N1	Mx
30	HG_Lch_50	High	57	6	5.43	T2c	Nx	Mx
31	HG_Lch_52	Low	60	7	5.1	T2b	N0	Mx
32	HG_Lch_53	VeryHigh	59	7	34.64	T3b	N1	Mx
33	HG_Lch_54	High	62	8	5.6	T2b	N0	Mx
34	HG_Lch_55	VeryHigh	57	10	15.95	T3b	N1	Mx
35	HG_Lch_56	High	50	6	5.7	T2c	Nx	Mx
36	HG_Lch_57	High	58	8	1.6	T2a	N0	Mx
37	HG_Lch_58	VeryHigh	60	8	9.28	T2b	N1	Mx
38	HG_Lch_59	High	65	7	9.21	T2R1	Nx	Mx

39	HG_Lch_60	Low	59	7	5.13	T2a	Nx	Mx
40	HG_Lch_61	High	64	7	4.87	T2c	Nx	Mx
41	HG_Lch_62	High	67	9	14.56	T2a	N0	Mx
42	HG_Lch_64	High	69	9	38.87	Tx	Nx	Mx

Supplemental Table S2. MBD-Seq data information

	Sample ID	Sample Type	Raw reads	Unique Mapped Reads	Recovered Multi-mapped Reads	Final Mapped Reads	Mapping Percentage
1	HG_LCH_01N	normal	37936734	24740715	10100582	34841297	91.84%
2	HG_LCH_04N	normal	40661558	26688329	11046502	37734831	92.80%
3	HG_LCH_05N	normal	54038752	30450618	18275423	48726041	90.17%
4	HG_LCH_06N	normal	49633325	32343426	13281013	45624439	91.92%
5	HG_LCH_09N	normal	44360027	30907913	10731777	41639690	93.87%
6	HG_LCH_10N	normal	52159226	33065040	14953560	48018600	92.06%
7	HG_LCH_11N	normal	50731967	31780016	14497846	46277862	91.22%
8	HG_LCH_12N	normal	50031476	32371385	13926639	46298024	92.54%
9	HG_LCH_21A	Low Adjacent	48195820	27947752	16570955	44518707	92.37%
10	HG_LCH_21T	Low	35883180	18722178	13644229	32366407	90.20%
11	HG_LCH_22A	Low Adjacent	55846770	35027289	16942145	51969434	93.06%
12	HG_LCH_22T	Low	51113317	32055208	15881497	47936705	93.79%
13	HG_LCH_23A	Low Adjacent	52258506	33915259	15098915	49014174	93.79%
14	HG_LCH_23T	Low	52815451	34775856	14810245	49586101	93.89%
15	HG_LCH_24A	Low Adjacent	52495902	32827680	15911211	48738891	92.84%
16	HG_LCH_25A	Low Adjacent	66772817	42035330	19763623	61798953	92.55%
17	HG_LCH_26A	Low Adjacent	44482565	27574153	13609480	41183633	92.58%
18	HG_LCH_26T	Low	34736522	16351404	14689580	31040984	89.36%
19	HG_LCH_27A	Low Adjacent	53546658	32892276	16225828	49118104	91.73%
20	HG_LCH_27T	Low	67669267	43907355	18595647	62503002	92.37%
21	HG_LCH_28T	Low	50491187	36524791	11013185	47537976	94.15%
22	HG_LCH_29A	Low Adjacent	46548956	30195594	13059088	43254682	92.92%
23	HG_LCH_29T	Low	31759354	19800894	9499959	29300853	92.26%
24	HG_LCH_30A	Low Adjacent	48733809	31608045	13679844	45287889	92.93%
25	HG_LCH_30T	Low	84008290	60500705	18326573	78827278	93.83%
26	HG_LCH_31A	Low Adjacent	52733407	35951055	13686286	49637341	94.13%
27	HG_LCH_31T	Low	46494014	31719929	12418218	44138147	94.93%
28	HG_LCH_32T	Low	55566715	38485297	14064408	52549705	94.57%
29	HG_LCH_35T	High	78833093	45058947	27772800	72831747	92.39%
30	HG_LCH_36T	High	47728652	27591043	16143671	43734714	91.63%
31	HG_LCH_38T	Very High	56826300	34793765	17693170	52486935	92.36%
32	HG_LCH_39T	Very High	198484398	97383590	82583807	179967397	90.67%
33	HG_LCH_42T	Very High	101485884	52014342	41237800	93252142	91.89%
34	HG_LCH_45A	High Adjacent	55620733	35459979	15362048	50822027	91.37%
35	HG_LCH_45T	High	42982106	21072185	16884711	37956896	88.31%
36	HG_LCH_47A	Low Adjacent	52363667	26757691	20199514	46957205	89.68%
37	HG_LCH_47T	Low	47052155	23319726	18689231	42008957	89.28%
38	HG_LCH_48T	Very High	37223159	19166383	14707875	33874258	91.00%

39	HG_LCH_49T	High	143507709	73794267	57228721	131022988	91.30%
40	HG_LCH_50T	High	27749151	13538390	11466398	25004788	90.11%
41	HG_LCH_52T	Low	35635992	16905961	12288701	29194662	81.92%
42	HG_LCH_53T	Very High	34600126	17431557	13992667	31424224	90.82%
43	HG_LCH_54T	High	35020773	17091101	14780620	31871721	91.01%
44	HG_LCH_55T	Very High	53434859	38714549	11472466	50187015	93.92%
45	HG_LCH_56T	High	35398587	17400712	14557720	31958432	90.28%
46	HG_LCH_57T	High	40778665	19272863	17354738	36627601	89.82%
47	HG_LCH_58T	Very High	52214996	36043811	12442761	48486572	92.86%
48	HG_LCH_59T	High	54693875	26539670	21555528	48095198	87.94%
49	HG_LCH_60T	Low	35032737	14111989	10647664	24759653	70.68%
50	HG_LCH_61T	High	45019969	22491961	18529350	41021311	91.12%
51	HG_LCH_62T	High	35856797	17330056	14683016	32013072	89.28%
52	HG_LCH_64T	High	57837869	41523842	12749045	54272887	93.84%

Supplemental Table S3. Number of genes used in IPA analysis

CDMP	Up-regulated Genes	Down-regulated Genes
S1D1	1210	1505
S1D0	102	163
S0D1	27	11
S0D0	57	37
S1G1	524	653
S1E1	692	652
S0E0	22	10

Supplemental Table S4. Genes shown in the network in Figure 4 that are also up regulated in TCGA data

Gene	Gene	Gene	Gene
ABCG1	EZH2	MAD2L2	SERPINB6
AURKA	F2	MMP9	SMARCA4
BAX	FBP1	NDUFA13	STC2
BIRC5	FEN1	NPM1	TAP2
CCNE1	FGF8	NPR3	TARBP2
CDC25A	FKBP4	PDIA3	TCF3
CDK5	FOXRED2	PKMYT1	TERT
CDKN2A	HEYL	PKN1	TGM2
CDT1	HK2	PSMB5	TOP2A
CLGN	IER2	PSMB7	TPX2
CYP1A1	IGF2R	PTK6	TRADD
CYP1B1	IL11	PYCR1	TYMP
DGAT2	KCNJ12	RAD9A	TYMS
DNMT3B	KIF15	RNF126	WDR5
E2F1	KIF2C	SERF1A/SERF1B	

Supplemental Table S5. Genes shown in the network in Figure 4 that are also down regulated in TCGA data

Gene	Gene	Gene	Gene
ADRA1D	CXCL12	KDR	PTGS2
ANGPT1	CYP24A1	KIT	PTN
AXL	DAB2IP	KLF4	RGS12
BMP4	DIRAS3	KLF6	RNF128
BMP7	DNAJC15	LAMB3	SCK1
BST1	EDN2	LOC102724428/SIK1	SDC1
CAV1	EGFR	LOXL2	SDC3
CCNA1	ENO2	MET	SGK1
CCNT1	ENPP2	MYL9	SLC22A17
CD34	EPAS1	NGF	SMAD3
CD9	GLI2	NOTCH1	SMAD7
CNTN1	GSTM1	PCOLCE2	SPARC
COL4A5	GSTM2	PDGFRA	SV2A
COL4A6	HMGA2	PDK4	TAGLN
COL5A1	IGFBP3	PEG10	TP73
COL6A1	IGFBP4	PHLDA1	TRIP6
COL6A2	IRF5	PHLDA3	USP2
COL6A3	IRS1	PLAU	VIM
CRISPLD2	ITGA3	PPP1R15A	WNT3A
CRYAB	ITGA5	PRKCE	WNT5A

Supplemental Table S6. Genes that are only in very high risk group and also differentially methylated in cohort as well as differentially expressed in TCGA RNA-seq data

CMP	No.	Gene
S0D0	73	ACAP1, AIFM1, APLN, ARMCX1, ATP6AP1, BAGE, BLK, BRS3, CDH4, CDK10, CLDN2, CLEC4C, DEGS1, EP400NL, FAM66A, FAM86B2, FBP1, FMR1NB, GDF3, GLT6D1, GTF2H2B, GTSE1, GUSBP3, HMGB3, HSH2D, IGSF22, IL9R, L1CAM, LAIR2, LDHB, LOC100134868, LOC440896, LOC647859, MAP2K2, MAP7D3, MEF2B, MUC20, MYH3, NAA10, NLRP8, NVL, NXF3, PAK3, PLAC1, PNCK, PNMA5, PNPLA5, PPAN, PPP1R14A, PRR5, PRR5-ARHGAP8, PRY2, RPL39, RUVBL2, SERF1A, SLC25A45, SLCO1A2, SMYD4, SNRNP70, SPATA2L, SSX2, SURF2, SYTL4, TAF7L, TECPR1, TK1, TKTL1, TUSC5, UPK1A, XKR5, XKRX, XPNPEP2, ZNF423
S0D1	16	AFMID, AIFM1, ATP6AP1, CDK10, HSH2D, IKZF1, NOP56, NOS3, NVL, PPAN, RUVBL2, SIGLEC11, SLC25A45, SPATC1, UPK1A, ZNF732
S1D0	82	ABCC3, ADCYAP1R1, ANO5, BEGAIN, C1QTNF1, CA3, CAMK1D, CIDEA, CLDN10, CPEB1, CPNE7, CYBA, DAAM2, DDX25, DNAH10, DPYSL2, EFEMP2, ELF4, EMX1, EPS8L1, FAM69C, FBLN5, FLRT2, FNDC1, FOSL1, FOXF2, GALR1, GAS7, GATA4, GFPT2, GRIK3, GRIN2A, HCG11, HFE, IKZF1, ITPKA, KCNH2, KCNJ12, KCTD14, KLHDC8A, KLHL21, LHX8, LINGO1, LYPD1, MAP1B, MAPK15, MDGA2, MFI2, MMP2, MMP28, MSX1, NEU1, NRN1, NUAK1, PFKP, PITX2, PRRG3, PYGL, RGMA, RNF128, SHC3, SLC16A5, SLC17A9, SLC1A6, SOX1, SPATS1, SYT15, SYT9, TBX20, TFCP2L1, TIMP2, TRIP6, UCKL1, VGLL2, VIPR2, VSTM2L, WNT3A, WWC2, ZIC3, ZNF154, ZNF655, ZNF853
S0G0	14	DAZ2, DAZ4, EIF3C, FAM66A, FAM86B2, GAGE1, GTF2H2B, HSH2D, MEF2B, NBPF15, NVL, RUVBL2, SYTL4, TUSC5
S0E0	25	AIFM1, CBWD5, CELF5, DEGS1, EIF3C, FAM66A, FAM86B2, FBP1, GCNT1, GLT6D1, GTF2H2B, HMGB3, HSH2D, IL9R, LAIR2, LOC100134868, MUC20, MYH3, NVL, NXF3, SERF1A, SSH1, SSX2, TAF7L, XPNPEP2
S1E0	9	FGF14, GRM6, LINGO2, NRK, ST8SIA1, STK32B, SV2A, TIMP2, TMEM132C

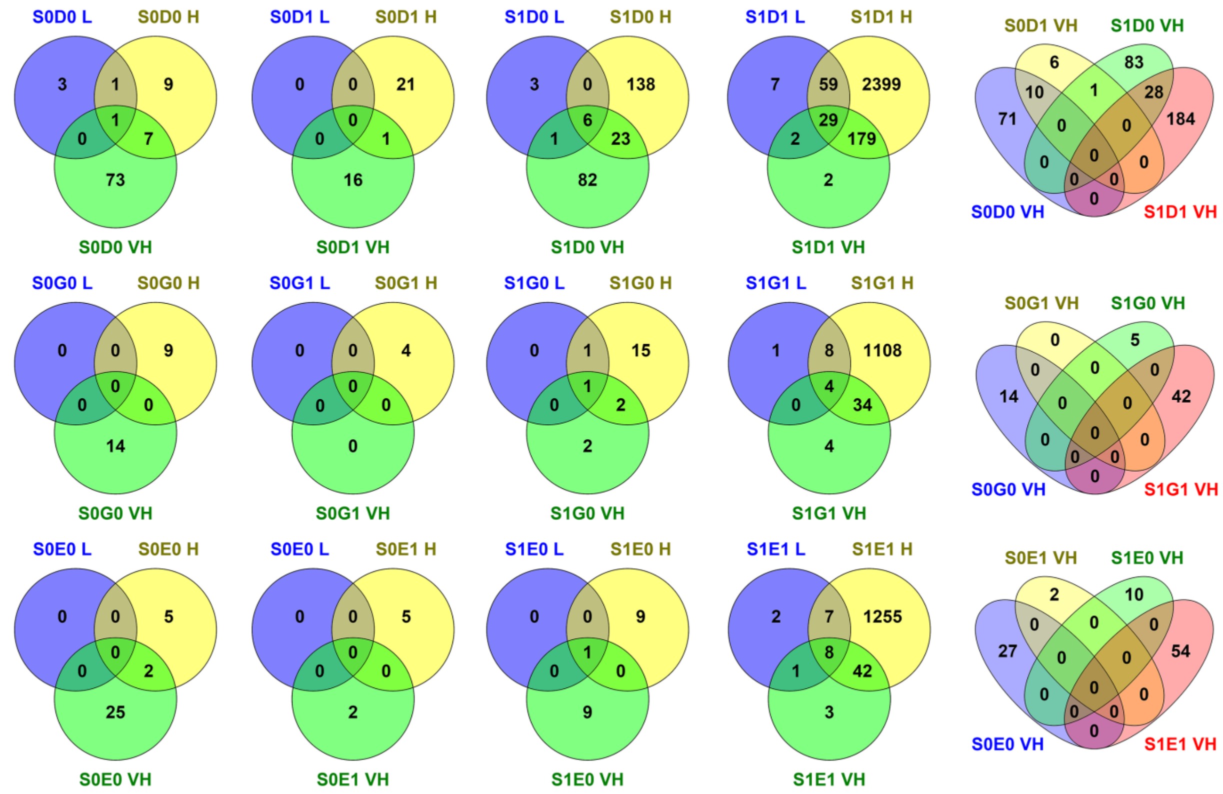

Supplemental Figure S1. Venn diagram of differential expressed genes between different risk group in each CMP

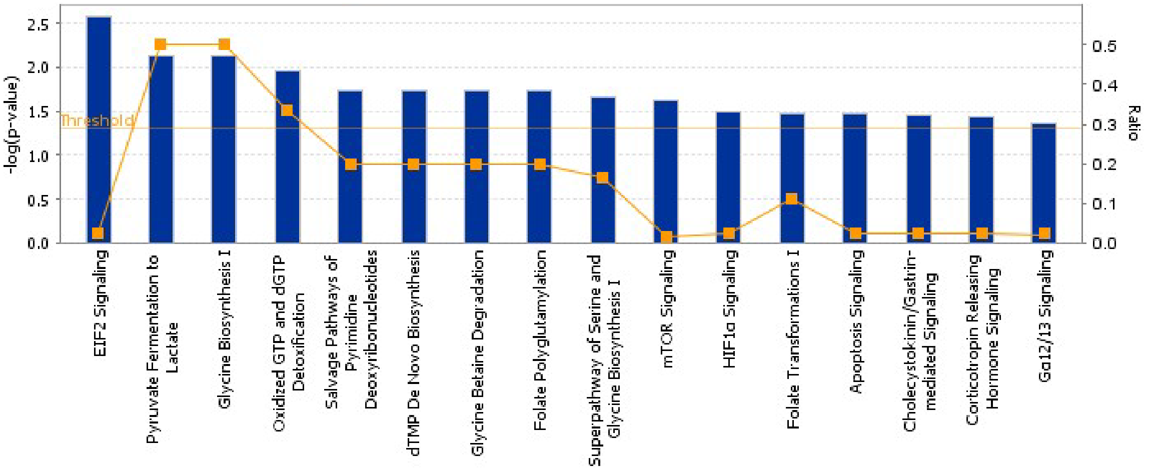

Supplemental Figure S2. S0D0 Canonical Pathways

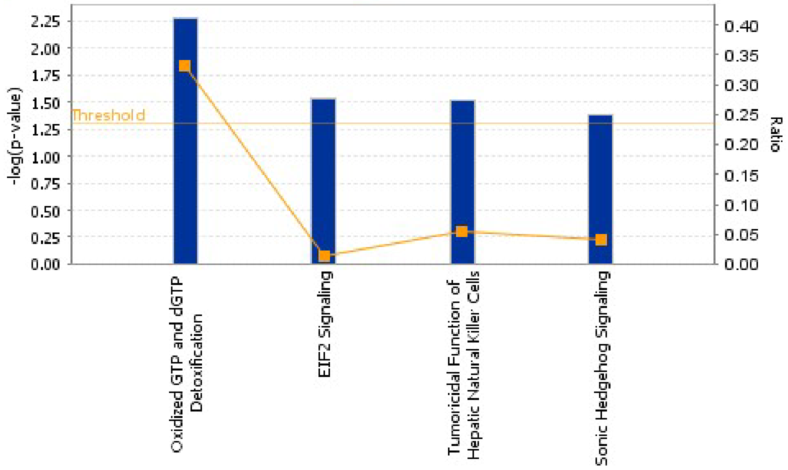

Supplemental Figure S3. S0D1 Canonical Pathways

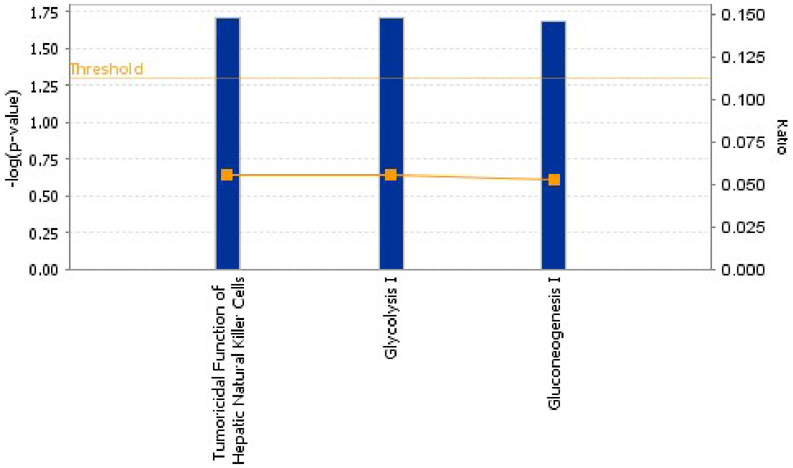

Supplemental Figure S4. S0E0 Canonical Pathways

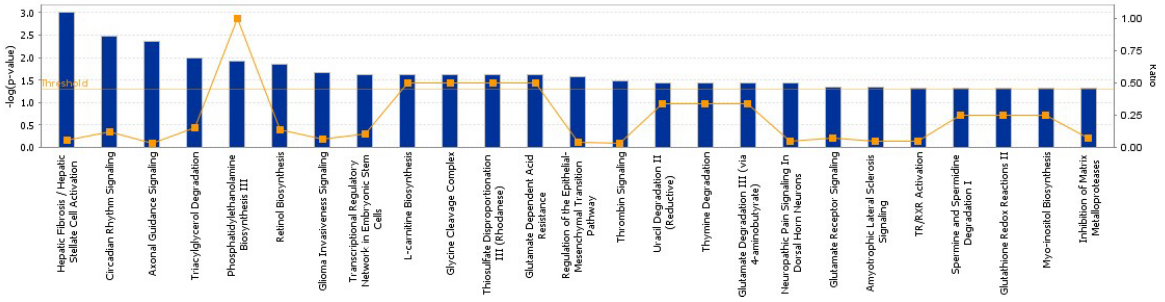

Supplemental Figure S5. S1D0 Canonical Pathways

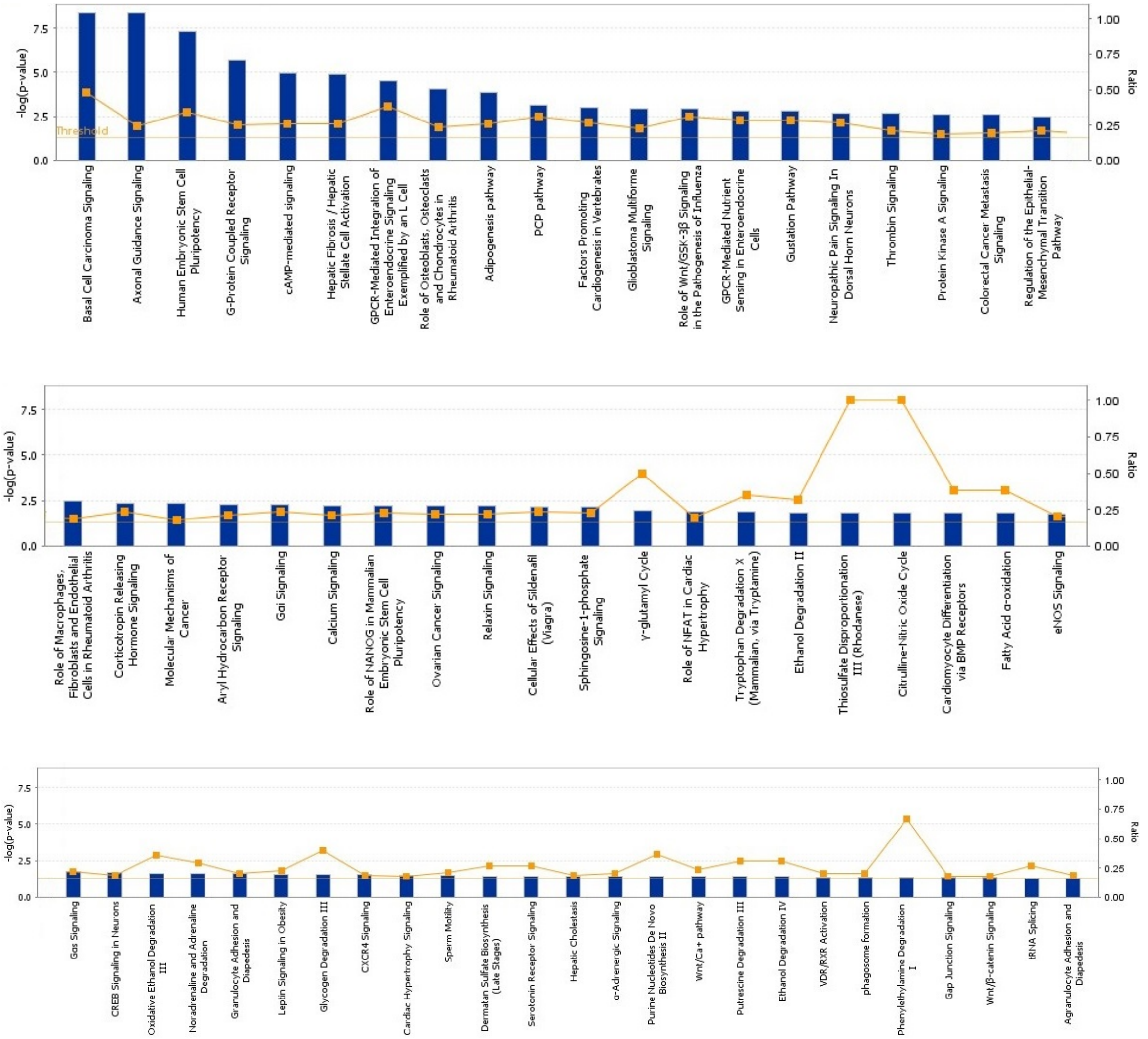

Supplemental Figure S6. S1D1 Canonical Pathways

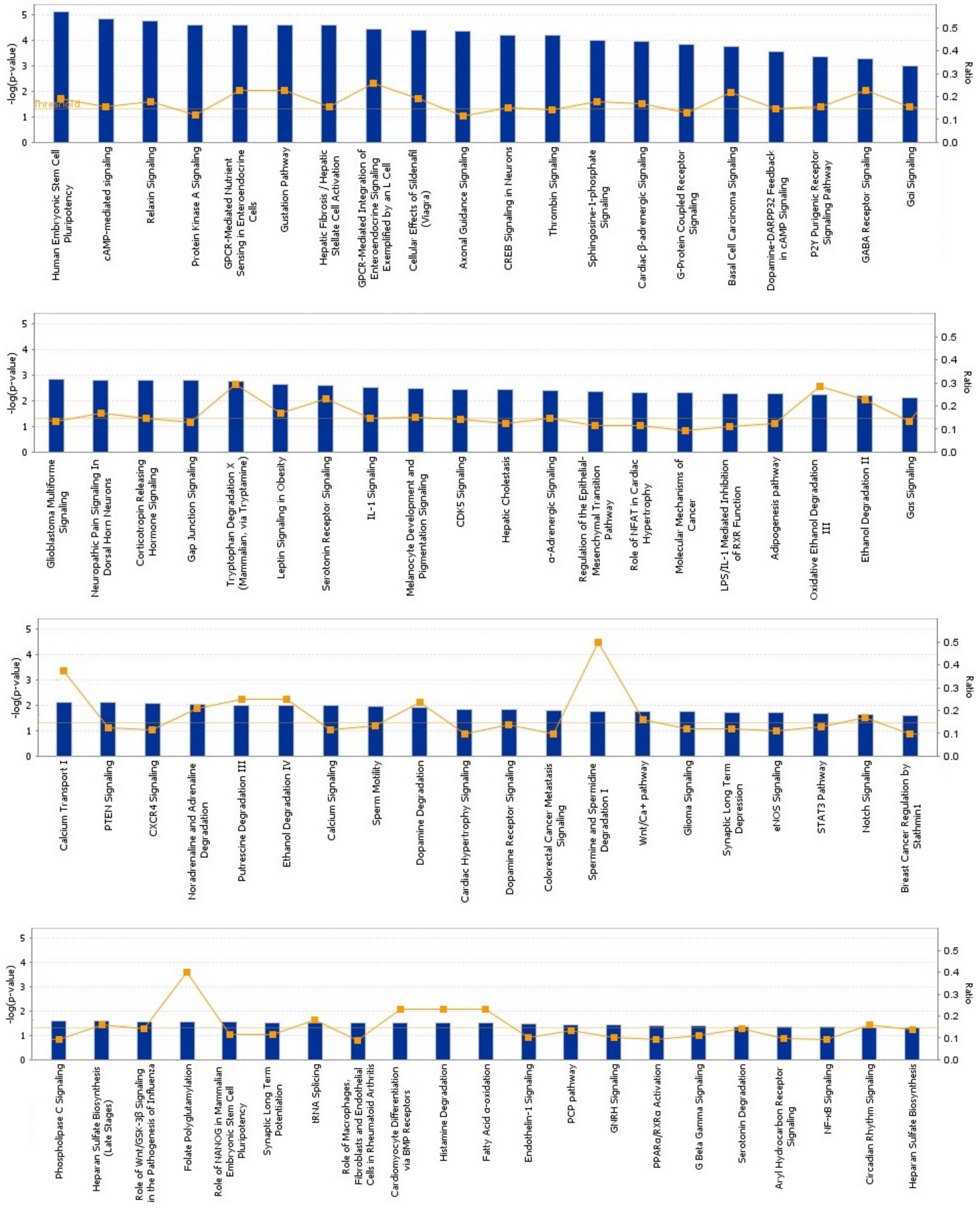

Supplemental Figure S7. S1G1 Canonical Pathways

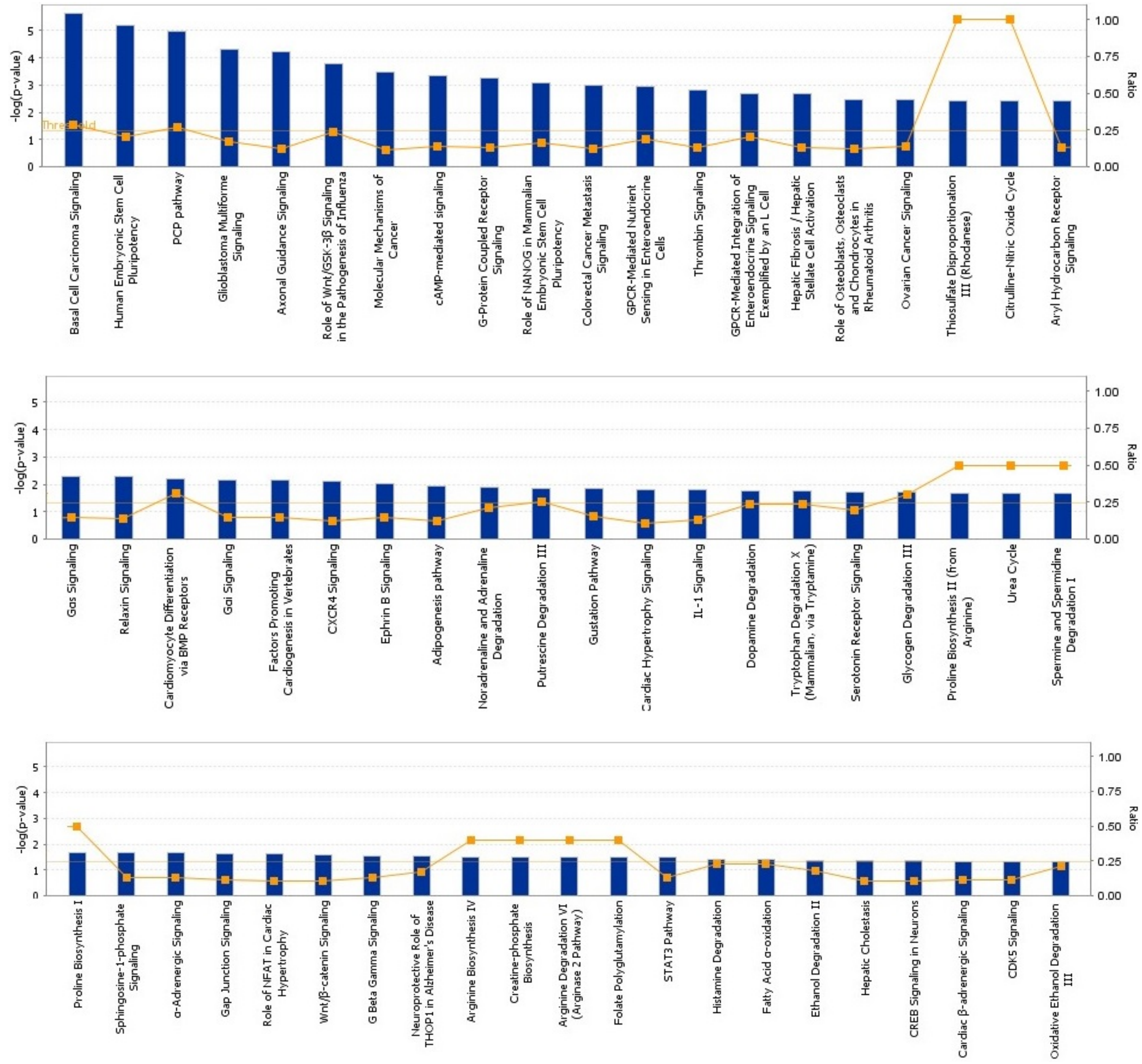

Supplemental Figure S8. S1E1 Canonical Pathways

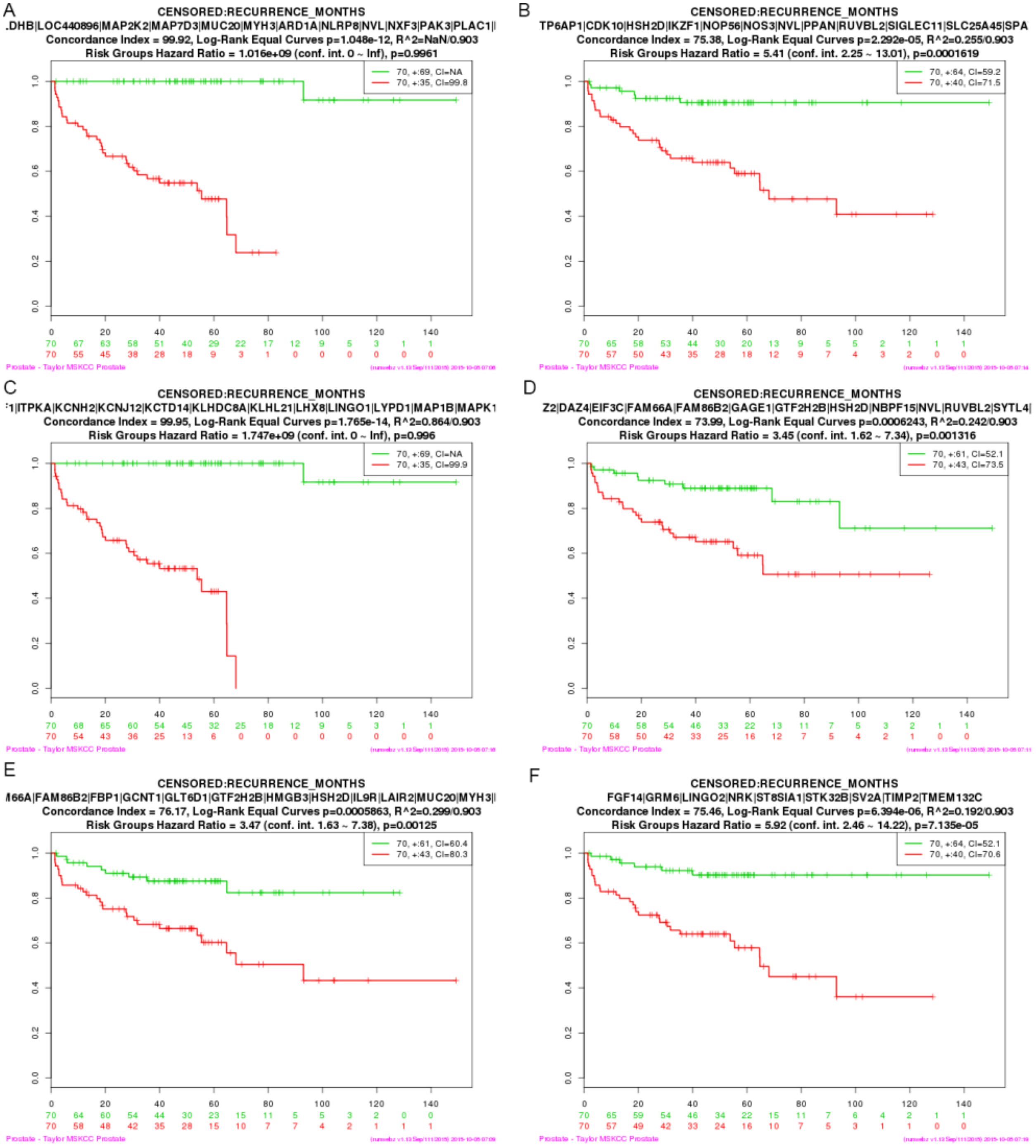

Supplemental Figure S9. Kaplan Meier curve of genes that are only differentially expressed and methylated in very high risk group
A: S0D0, B: S0D1, C: S1D0, D: S0G0, E: S0E0, F: S1E0.
